# Novel prime-boost immune-based therapy inhibiting both hepatitis B and D virus infections

**DOI:** 10.1136/gutjnl-2022-327216

**Published:** 2022-08-17

**Authors:** Rani Burm, Panagiota Maravelia, Gustaf Ahlen, Sandra Ciesek, Noelia Caro Perez, Anna Pasetto, Stephan Urban, Freya Van Houtte, Lieven Verhoye, Heiner Wedemeyer, Magnus Johansson, Lars Frelin, Matti Sällberg, Philip Meuleman

**Affiliations:** 1 Laboratory of Liver Infectious Diseases (LLID), Department of Diagnostic Sciences, Faculty of Medicine and Health Sciences, Ghent University, Ghent, Belgium; 2 Division of Clinical Microbiology, Department of Laboratory Medicine, Karolinska Institutet, Stockholm, Sweden; 3 Institute for Medical Virology, University Hospital, Goethe University, Frankfurt am Main, Germany; 4 Fraunhofer Institute for Translational Medicine and Pharmacology ITMP, Frankfurt am Main, Germany; 5 German Center for Infection Research, DZIF, External partner site, Frankfurt am Main, Germany; 6 Department of Infectious Diseases, Molecular Virology, University Hospital Heidelberg, Heidelberg, Germany; 7 Department of Gastroenterology, Hepatology and Endocrinology, Hannover Medical School, Hannover, Germany; 8 School of Medical Sciences, Inflammatory Response and Infection Susceptibility Centre (iRiSC), Faculty of Medicine and Health, Örebro University, Orebro, Sweden

**Keywords:** antiviral therapy, hepatitis B, hepatitis D, immunotherapy, chronic viral hepatitis

## Abstract

**Objective:**

Chronic HBV/HDV infections are a major cause of liver cancer. Current treatments can only rarely eliminate HBV and HDV. Our previously developed preS1-HDAg immunotherapy could induce neutralising antibodies to HBV in vivo and raise HBV/HDV-specific T-cells. Here, we further investigate if a heterologous prime-boost strategy can circumvent T-cell tolerance and preclude HDV superinfection in vivo.

**Design:**

A DNA prime-protein boost strategy was evaluated for immunogenicity in mice and rabbits. Its ability to circumvent T-cell tolerance was assessed in immunocompetent hepatitis B surface antigen (HBsAg)-transgenic mice. Neutralisation of HBV and HDV was evaluated both in vitro and in immunodeficient human-liver chimeric mice upon adoptive transfer.

**Results:**

The prime-boost strategy elicits robust HBV/HDV-specific T-cells and preS1-antibodies that can effectively prevent HBV and HDV (co-)infection in vitro and in vivo. In a mouse model representing the chronic HBsAg carrier state, active immunisation primes high levels of preS1-antibodies and HDAg-specific T-cells. Moreover, transfer of vaccine-induced antibodies completely protects HBV-infected human-liver chimeric mice from HDV superinfection.

**Conclusion:**

The herein described preS1-HDAg immunotherapy is shown to be immunogenic and vaccine-induced antibodies are highly effective at preventing HBV and HDV (super)infection both in vitro and in vivo. Our vaccine can complement current and future therapies for the control of chronic HBV and HDV infection.

**WHAT IS ALREADY KNOWN ON THIS SUBJECT?**

- Currently, there is no therapy available to eliminate chronic HBV or HDV infections.
- Chronic HDV infection is the most harmful among all viral hepatitis infections with the highest mortality rates.
- Involvement and boosting of the host innate and adaptive immune responses are required to circumvent immune evasion strategies exerted by HBV and to achieve a highly demanded functional cure.
- We previously developed a preS1-HDAg immunotherapy that provides HBV/HDV-specific T-cells and neutralising antibodies to HBV in vivo.

**WHAT ARE THE NEW FINDINGS?**

- This vaccine approach is able to prevent both HBV and HDV infection in vitro with a clear dose-response profile.
- Moreover, transfer of immunotherapy-induced antibodies from immunocompetent vaccinated animals can effectively prevent HBV/HDV co-infection and, more importantly, prevent HDV superinfection in the human-liver chimeric mouse model.
- Active immunisation of hepatitis B surface antigen (HBsAg)-transgenic mice, representing the chronic HBsAg carrier state, is able to induce high levels of preS1-antibodies and HDAg-specific T-cells.

**HOW MIGHT IT IMPACT ON CLINICAL PRACTICE IN THE FORESEEABLE FUTURE?**

- This vaccine approach is able to circumvent the problematic HBsAg-specific T-cell tolerance and is able to promote preS1-antibody production in a chronically infected host setting.
- Our strategy might efficiently complement existing or emerging therapies that block viral maturation to finally achieve a functional cure for HBV and HDV or to prevent chronic HBV-infected patients from acquiring a detrimental HDV superinfection.

## Introduction

Hepatitis D virus (HDV) infection in patients chronically infected with hepatitis B virus (HBV) represents the most severe form of all viral *hepatitides* causing the highest mortality rates.^1^ HDV requires coating with HBV envelope proteins, namely hepatitis B surface antigens (HBsAg), for its assembly and subsequent viral spread.^2–4^ Despite the availability of a preventive HBV vaccine, yet over 250 million people are currently chronically infected and about 5%–13% of these (up to 60 million people) will ultimately acquire an HDV infection.^5 6^ If HDV persists, the risk for developing hepatocellular carcinoma (HCC) increases by threefold compared with HBV mono-infection within 5–10 years after diagnosis.^1 7^

Currently, there is no effective functional cure for either chronic HBV or HDV. For patients with chronic HDV, standard therapy remains pegylated-interferon-α, with infrequent long-term responses.^8–11^ A recent clinical advancement for HDV treatment focuses on inhibiting viral entry through disrupting the binding complex between the preS1 domain of the large HBsAg (L-HBsAg) protein of HBV/HDV with the Na^+^-taurocholate co-transporting polypeptide (NTCP) receptor on the hepatocyte surface.^12^ In 2020, the peptide-based entry-inhibitor Myrcludex B (bulevirtide) received conditional marketing approval for treatment of patients with chronic HDV in Europe.^13^ Although encouraging clinical outcomes, the possibility to achieve sustained off-therapy response requires additional monitoring due to HBsAg persistence. Moreover, different (immune-based) vaccine strategies have been tested in mice and the woodchuck (*Marmota monax*) model using woodchuck hepatitis virus (WHV, an orthohepadnavirus showing strong homology with HBV), however with limited therapeutic outcomes so far.^14–24^

Current HBV therapy comprises lifelong administration of nucleos(t)ide analogues that can effectively suppress viral replication, however blocking of reverse transcription neither affects protein production and release (especially of HBsAg), nor synthesis of the covalently closed circular DNA, both of which represent the main causes of HBV persistence and consequently HCC development.^13 25 26^

The importance of host innate and adaptive immunity in controlling HBV infection is currently widely accepted.^27–29^ However, the major challenge remains circumventing the immune dysfunction and tolerance that is caused by multiple HBV immune escape mechanisms, especially extensive overproduction of subviral, non-infectious particles that sequester S-specific neutralising antibodies induced by traditional S-based HBV vaccines.^25 30–33^

These facts highlight the need for a finite treatment that achieves long-term control and ultimately a functional cure for both infections.^34^ This will likely require a combined therapy approach targeting different steps of the viral life cycle and at the same time boosting host immunity to clear the infected hepatocyte reservoir and/or prevent infection of naïve hepatocytes.

Recently, we developed a novel immunotherapeutic strategy able to prevent HBV mono-infection in human-liver chimeric mice.^35^ This immunotherapy is based on preS1-sequences to induce preS1 entry-inhibiting antibodies that can be more efficiently directed towards infectious virions compared with antibodies targeting S-HBsAg and M-HBsAg.^31 36–38^ The preS1-sequences are linked to HDAg, where the latter acts as a heterologous T-cell epitope able to prime naïve T-cells towards preS1.^35^ More specifically, although HBV-specific T-cell immunity is impaired in chronic HBV mono-infected patients, the HDAg epitope can still prime naïve T-cells to assist in B-cell maturation for endogenous antibody production and induction of T-cells towards both counterparts (preS1 and HDAg) able to kill infected hepatocytes. Next to bypassing this HBV-induced tolerance, the boosted adaptive immune response may also protect these patients from acquiring HDV superinfection.

**Figure 1:**
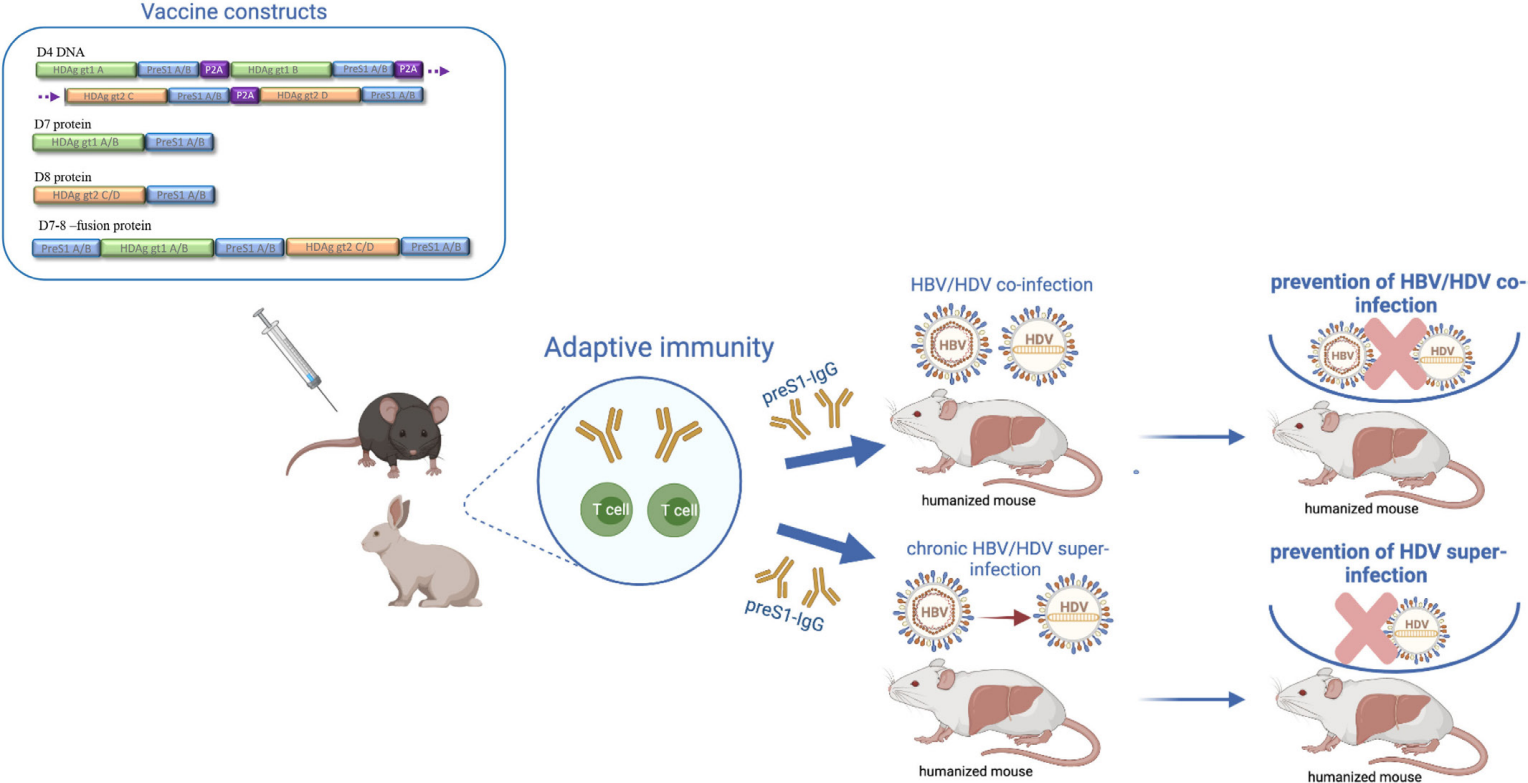
Vaccine constructs and experimental overview. Four different DNA/protein preS1-HDAg fusion constructs were evaluated for immunogenicity in mice and rabbits based on a heterologous (DNA prime-protein boost) or homologous (DNA prime-boost or protein prime-boost) vaccination strategy in order to assess adaptive immune responses (T-cells and antibodies). The vaccine-induced preS1-antibodies were further evaluated for their ability to neutralise HBV and HDV both in a co-infection and superinfection setting in homozygous urokinase-type plasminogen activator-severe combined immunodeficiency (uPA^+/+^-SCID) human-liver chimeric mice. Image was created with BioRender.com.

In this study, we first assessed whether a heterologous DNA prime-protein boost strategy could efficiently enhance HBV/HDV-specific T-cells and entry-inhibiting preS1-antibodies in wild-type mice. We next seeked to investigate whether active immunisation of immunocompetent HBsAg-transgenic (HBsAg-Tg) mice, representing the chronic HBsAg carrier state, could trigger T-cell and antibody responses.^39^ Finally, we evaluated if adaptive transfer of vaccine-induced preS1-antibodies could prevent HBV/HDV co-infection and superinfection in vitro and in immunodeficient human-liver chimeric mice (figure 1).

## Materials and methods

A more detailed version of this section can be found in online supplemental materials.

10.1136/gutjnl-2022-327216.supp1 Supplementary data

### Synthesis of preS1-HDAg vaccine constructs and immunisation protocols

All D4, D7 and D8 vaccine constructs harbour different combinations of L-HDAg (gt1 and 2) and preS1A/B consensus sequences (amino acids 2–48) (figure 1). The HDAg sequences were obtained from four different clinical isolates: US-2 and CB; and 7/18/83 and TW2476. D4 DNA construct generation and evaluation were conducted exactly as previously described.^35^ For the three recombinant proteins, namely D7, D8 and D7-D8 fusion protein constructs, the vector pET-30a(+) with His-tag (C-terminal) was used with the *Escherichia coli* BL21 Star (DE3) expression system. All produced protein preparations have passed quality control and were assessed for endotoxin removal and purity. Endotoxin levels were quantified as 2.6 EU/mg (D7 protein), 0.73 EU/mg (D8 protein) and below 100 EU/mg (D7-D8 fusion protein construct). Synthesis, subcloning, upscale production, protein purification and expression analysis were conducted by GenScript (online supplemental material and figure 1).

10.1136/gutjnl-2022-327216.supp2 Supplementary data

Immunisations in wild-type C57BL/6J or HBsAg-Tg mice and rabbits using plasmid DNA were conducted as previously.^35^ D7 and D8 protein preparations were administered subcutaneously either individually (10 µg), as a mix (20 µg) or as fusion protein (20 µg) (online supplemental table 1). Prior to identifying QS21 as a more immunogenic adjuvant (figure 3A), alum was used in some animal studies (figure 6 and online supplemental table 1). Extended information is available in the online supplemental file.

### Evaluation of prime-boost immunogenicity by ELISA and enzyme-linked immunospot assay

ELISA and enzyme-linked immunospot assay (ELISpot) assays were conducted exactly as has previously been described.^35 40^ Detailed description is available in the online supplemental file. Information on the peptides used in ELISpot assays is provided in online supplemental table 2.

### HBV/HDV viral inocula

HBV (gtD) was produced using the tetracycline-inducible cell line HepAD38.^41^ For HDV (gt1) production, plasmids pSVL(D3) and pT7HB2.7 were co-transfected in Huh7.5 cells. Supernatant of both was collected and concentrated using PEG precipitation. Plasma from HBV mono-infected and HBV/HDV co-infected patients was also used as viral inoculum for the in vivo neutralisation experiments and as primary antibody source for HDV-specific immunofluorescent stainings.

### In vitro neutralisation of HBV and HDV with mouse preS1-HDAg antiserum

For prevention of both HBV and HDV infection in vitro, HepG2.hNTCP cells were seeded in poly-L-lysine-coated 96-well plates. Three days later, HBV (2.530 IU/cell) or HDV inoculum (4 IU/cell) in complete Dulbecco’s Modified Eagle Medium (cDMEM) supplemented with 2.5% dimethylsulphoxide (DMSO) and 4% PEG8000 was incubated for 1 hour at room temperature with different dilutions of mouse antiserum (online supplemental table 1). Naïve serum was included as control and all conditions were performed in duplicate. This virus-antiserum mixture was subsequently added to the HepG2.hNTCP cells and incubated for 24 hours. Myrcludex B (MyrB, 200 nM) was used as control and applied to the cells 1 hour prior to virus incubation. After 24 hours, cells were washed three times with phosphate-buffered saline (PBS) to remove viral inoculum; and every 2–3 days, the antiserum/naïve mouse serum or MyrB was refreshed in cDMEM medium (with 2.5% DMSO) until read-out at day 8 postinfection.

### HBV and HDV immunofluorescence staining and imaging

For hepatitis B core antigen (HBcAg) staining, cells were fixed, permeabilised and blocked overnight at 4°C. Cells were stained with a combination of polyclonal rabbit HBc-antibody (DakoCytomation, REF-B0586) and an AlexaFluor 488-labelled goat antirabbit IgG secondary antibody (Invitrogen, REF-A11034). HDV immunofluorescence (IF) staining was performed in a similar way, using HDV patient EDTA-plasma as primary antibody and an AlexaFluor 488 goat antihuman IgG secondary antibody (Invitrogen, REF-A11013). Nuclei were counterstained with 4',6-diamidino-2-phenylindole (DAPI). Images were captured using a Leica TCS-SPE confocal microscope (20× objective). Per well, three random pictures were taken and all conditions were performed in duplicate (six random pictures per condition). Automated cell counting was performed using ImageJ software V.1.53c.

### Generation of human-liver chimeric uPA^+/+^-SCID mice and passive immunisations

Human-liver chimeric mice were generated by transplantation of approximately 10^6^ primary human hepatocytes (donor C342 from Corning, The Netherlands or donor L191501 from Lonza, Switzerland) into homozygous urokinase-type plasminogen activator-severe combined immunodeficiency (uPA^+/+^-SCID) mice as previously described.^42 43^ Human albumin quantification in mouse plasma (via ELISA) was used to assess the level of liver humanisation.

For passive immunisations, human-liver chimeric uPA^+/+^-SCID mice were intrasplenically injected with 100 µL total mouse/rabbit antiserum or 5.5 mg purified rabbit IgG 1 day prior to HBV/HDV co-infection (figure 4) or HDV superinfection (figure 6 and online supplemental figure 4). Additional purified IgG injections (100 µL-5.5 mg) were intraperitoneally administered at day 1, 4, 7, 11 and 14 following HDV superinfection in C342-transplanted mice (figure 6); while additional total mouse antiserum (50 µL) injections were done at day 1, 4 and 7 following HDV superinfection in L191501-transplanted mice (online supplemental figure 4). Control mice were either injected with naïve serum or PBS. A detailed overview of all experimental set-ups is shown in online supplemental table 1. For HBV/HDV co-infections, mice were injected intraperitoneally with 100 µL of viral inoculum consisting of 5×10^6^ IU HBV and 1.48×10^6^ IU HDV (both cell-culture derived). For HDV superinfection, mice were first infected with HBV (5×10^6^ IU cell culture-derived, or 10^6^ IU patient-derived) and 8 weeks later with HDV (2.55×10^5^ IU cell culture or patient-derived). Blood plasma was collected and viraemia was determined by RealStar HDV/HBV (RT-)qPCR (Altona Diagnostics, Germany) following total nucleic acid extraction (NucliSENS EasyMag, BioMérieux, France). Plasma preS1-antibody titres were determined by ELISA as described above.

10.1136/gutjnl-2022-327216.supp5 Supplementary data

## Results

### Intrinsic immunogenicity of homologous and heterologous prime-boost strategies

**Figure 2:**
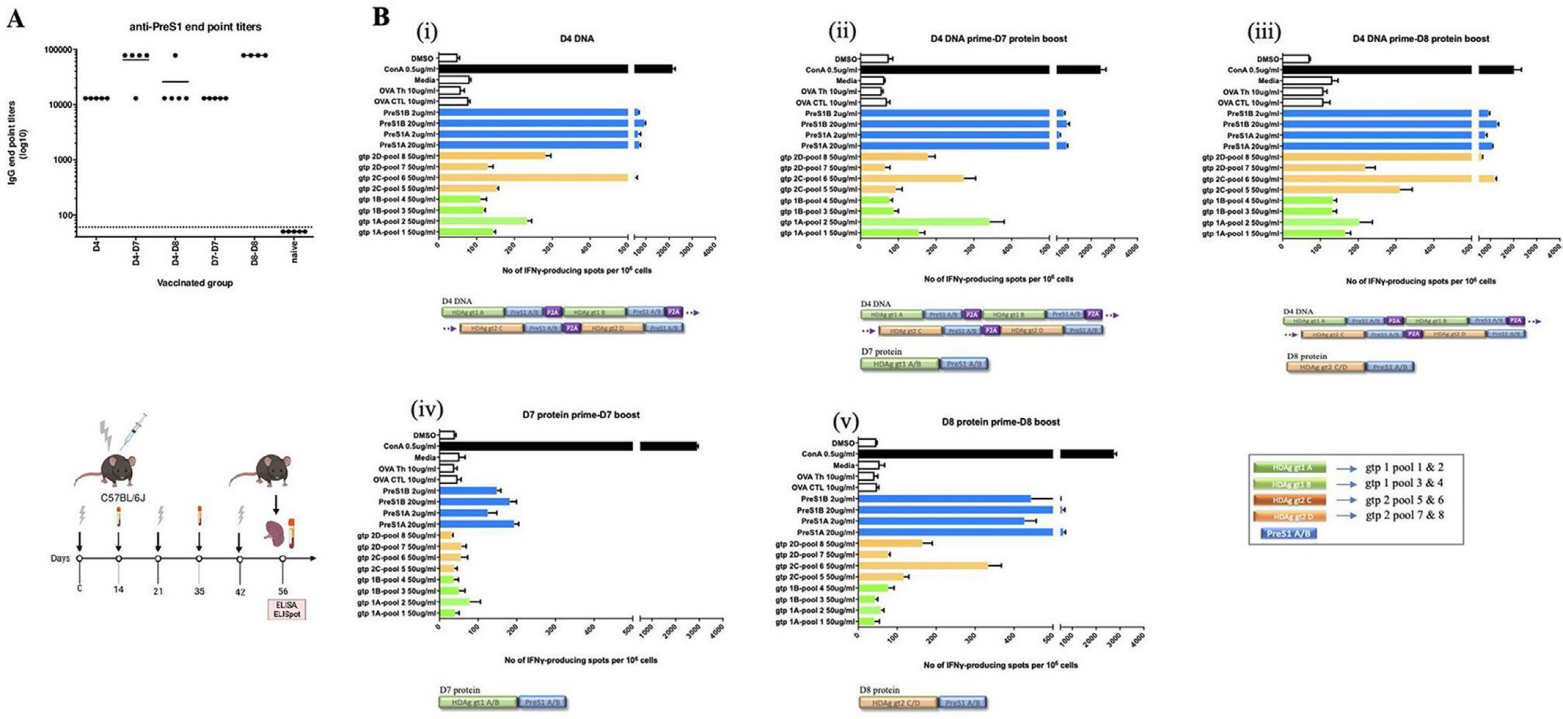
Induction of preS1-antibodies and HBV/HDV-specific T-cells following vaccination. C57BL/6J mice were primed with D4 DNA (50 µg) followed by in vivo electroporation and boosted twice at monthly intervals with D4 DNA, or D7 or D8 protein constructs, respectively (10 µg for each protein) or primed-boosted with D7/D8 proteins in incomplete Freud adjuvant. Two weeks after the final boost, mice were sacrificed to collect blood and splenocytes for subsequent ELISA (A) and interferon (IFN)-γ enzyme-linked immunospot assay (ELISpot) (B) analysis, respectively. (A) PreS1-antibody titres were determined as end point serum dilutions (at logarithmic scale) at which the optical density (OD) value at 405 nm was at least twice the OD of the negative control (naïve mouse serum) at the same dilution. The cut-off was set at 60, the starting dilution tested. Mean of five mice per vaccinated group is shown and each dot represents one mouse. (Bi–v) Induction of HBV/HDV-specific T-cells after 48 hours stimulation with indicated antigens. The D4 DNA prime-boost group was used as reference. Bars represent the mean number of IFN-γ spot-forming cells (SFCs) per million with SE from triplicate wells tested for each antigen condition. The different bar colours correspond to the respective sequences of the vaccine constructs, that is, green for HDAg gt1, orange for HDAg gt2 and blue for preS1A/B. HDV peptide pools 1–4 correspond to gt1 and pools 5–8 to HDAg gt2. Pools 1 and 2 of gt1 refer to sequence/isolate A, while pools 3 and 4 correspond to sequence/isolate B. Similarly for HDV gt2, pools 5 and 6 refer to sequence/isolate C and pools 7 and 8 to sequence/isolate D. Each pool contained 20 or 21 (for pools 1 and 5) 15-mer peptides with 10 amino acids (aa) overlap. PreS1A and preS1B peptides refer to the consensus sequences (total 47 aa; region 2–48 aa), while cytotoxic T-lymphocyte and T-helper ovalbumin peptides (respectively OVA CTL and OVA Th) at 10 µg/mL were used as negative controls. Concanavalin A (ConA) was used as assay positive control at 0,5 µg/mL. Image was created with BioRender.com.

We first determined the intrinsic immunogenicity of the various prime-boost strategies (figure 1 and online supplemental table 1). In brief, mice were primed intramuscularly with D4 DNA encoding preS1-HDAg fusion genes using in vivo electroporation or subcutaneously with individual D7 protein (HDV gt1 linked to preS1 sequences) and D8 protein (HDV gt2 linked to preS1) and boosted once or twice with D4 DNA, D7 protein, D8 protein or D7-D8 fusion protein constructs in adjuvant (online supplemental table 1). Mice were bled 2 weeks after each immunisation and sacrificed 2 weeks after the final boost whereafter blood and splenocytes from each group were collected for assessment of respectively preS1-antibody induction via ELISA (figure 2A) and IFN-γ production after 48 hours recall antigen stimulation in vitro with HBV and HDV peptides via ELISpot (figure 2B).

Regarding preS1-antibody induction, we observed that either the prime-boost with homologous preS1-HDAg proteins (D7 prime-D7 boost or D8 prime-D8 boost) in adjuvant alone, or the heterologous D4 DNA prime-(D7 or D8) protein boost strategy consistently induced 10^4^–10^5^ preS1-titres (figure 2A). Conceivably, priming with DNA encoding the preS1-HDAg combination followed by boosting with proteins encoding HDAg only (ie, D9 or D10), or prime-boosting with these sole HDAg proteins, limited the induction of preS1-antibodies or did not boost them at all (levels similar to naïve group), respectively, since these HDAg protein constructs do not contain any preS1-sequences (online supplemental figure 2A). Moreover, a clear relation between the preS1-antibody levels based on ELISA (online supplemental figure 2A) and the ability of each construct harbouring preS1 to neutralise HBV in vitro could be observed (online supplemental figure 2B). As such, both the DNA prime-protein boost and the homologous D7-D7 or D8-D8 protein prime-boost strategy consistently induced 50% virus neutralisation (VNT_50_) at levels >10^3^.

10.1136/gutjnl-2022-327216.supp3 Supplementary data

Regarding the T-cell responses towards preS1 and HDAg, the most potent responses were evoked by the heterologous DNA prime-protein boost strategy (figure 2Bii, iii and online supplemental figure 3). The homologous prime-boost regimens (i.e., D4 DNA prime-protein boost and D7-D7 or D8-D8 homologous protein prime-boost) primed IFN-γ-producing T-cells mainly to the preS1 and HDAg gt2 components (figure 2Bi, iv, v). Taken together, the DNA prime-protein boost strategy effectively induces broad T-cell responses to preS1 and HDAg antigens in a genotype-specific manner.

10.1136/gutjnl-2022-327216.supp4 Supplementary data

### Pres1-HDAg antisera from vaccinated mice neutralise HBV and HDV in vitro.

**Figure 3:**
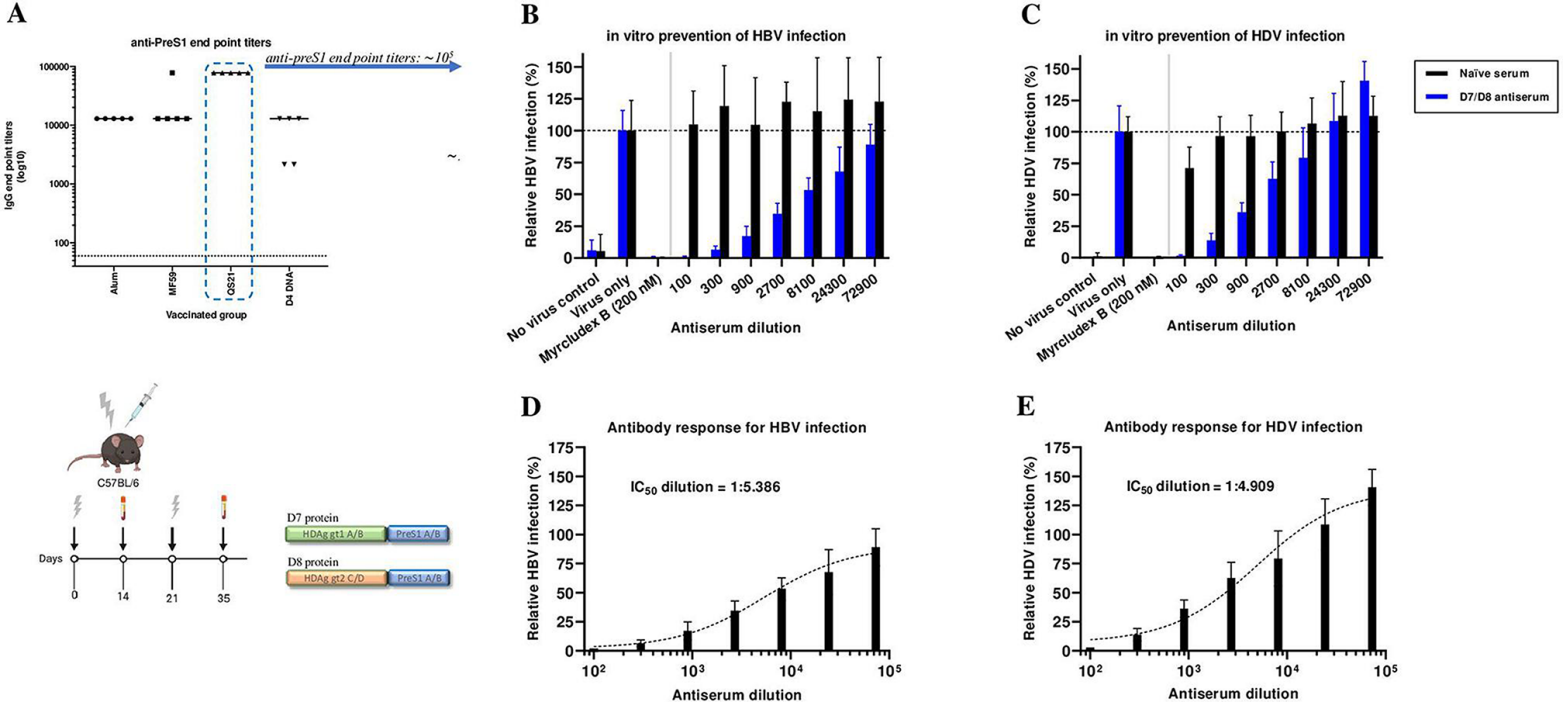
Prevention of HBV and HDV infection in vitro. C57BL/6 mice were primed and boosted once with D7/D8 protein mix in different adjuvants (alum, MF59 and QS21). Two weeks after the last boost, serum was collected and tested for preS1 end-titres based on ELISA. The D4 DNA prime-boost group was used as reference. (A) Mean anti-preS1 end point serum dilution logarithmic scale for each vaccinated group; each symbol represents one mouse. For prevention of in vitro HBV and HDV infection, mouse sera from the preS1-HDAg protein group (D7/D8) in QS21 adjuvant was used. HepG2.hNTCP cells were incubated with a mixture of pre-incubated cell culture-derived viral inoculum and mouse preS1-HDAg antiserum (blue) or naïve mouse serum (black). Antiserum or naïve mouse serum was applied as a 1:100 dilution and 1:3 serial dilutions in culture medium. Immunofluorescence staining was performed for cells after 1 week for HBcAg (B) and HDAg (C). (B–C) HBV and HDV infection, respectively (% relative to control condition ‘infection’ which is set to an arbitrary level of 100%). (D–E) Non-linear regression modelling and IC_50_ dilution for respectively HBV and HDV. All conditions were performed in duplicate and three images were captured per well (20×) using confocal microscopy. Infected cells were assessed using automatic cell counting (ImageJ software V.1.53c). Image was created with BioRender.com.

To evaluate whether vaccine-induced antisera could neutralise both HBV and HDV infection in vitro, antisera with preS1-titres close to 10^5^ (end point serum dilution on a log scale) derived from mice vaccinated twice with preS1-HDAg protein mix D7/D8 (figure 3A) were incubated with virus and then added to HepG2.hNTCP cells. One week later, cells were stained for HBcAg or HDAg to determine inhibition of infection. The lowest serum dilution tested (1:100) provided complete protection against both HBV and HDV (figure 3B, C), similar to MyrB at 200 nM. Serial threefold dilutions of the serum revealed a clear dose-response with half maximal inhibitory concentration (IC_50_) titres of 1:5.386 and 1:4.909 for HBV and HDV, respectively (figure 3D, E).

### Pres1-HDAg antisera from vaccinated mice prevent HBV/HDV co-infection in vivo.

**Figure 4:**
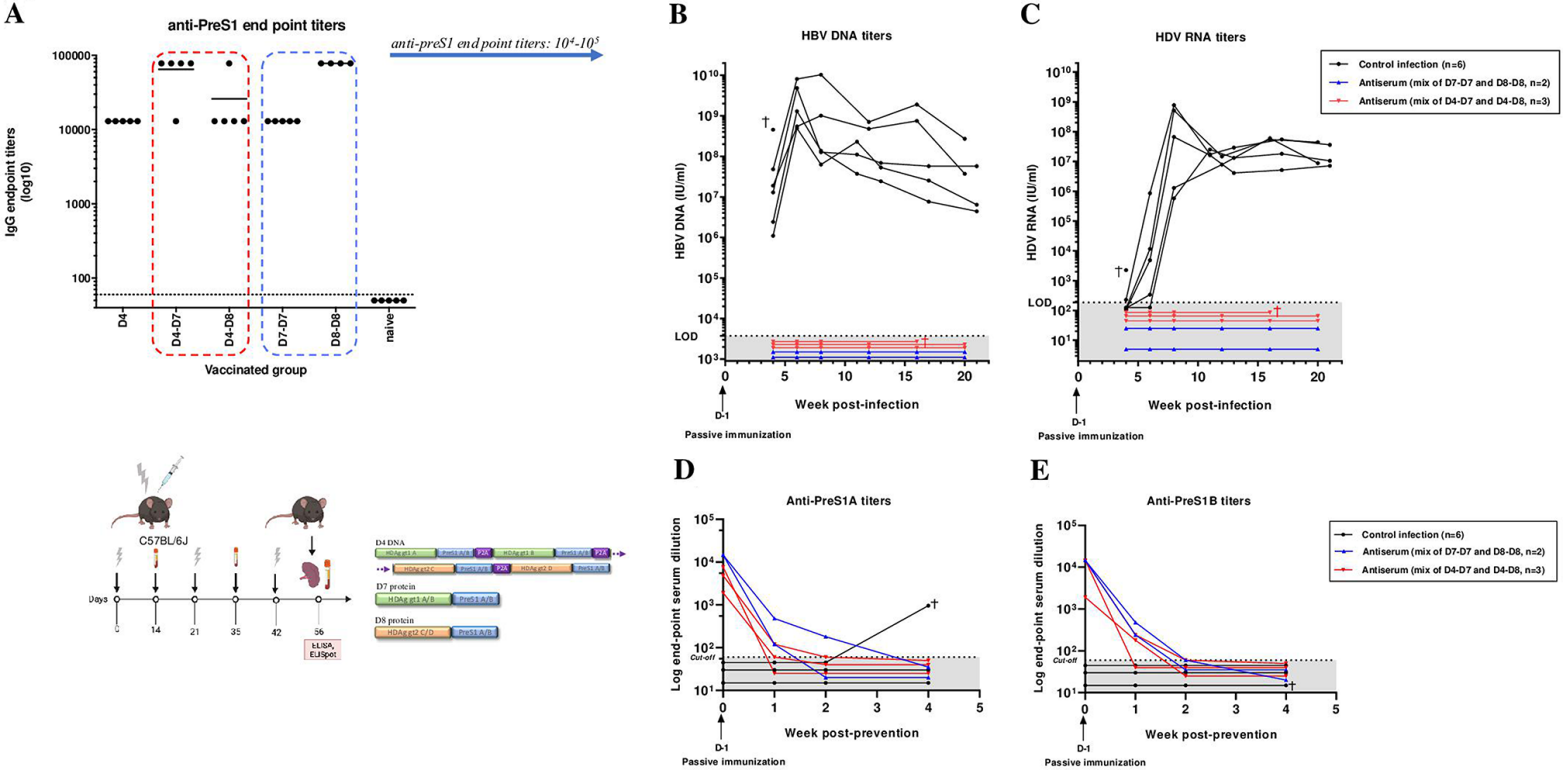
Prevention of HBV/HDV co-infection in vivo. Mouse antisera with titres ≥10^4^ (A) were passively administered through intrasplenical injection in human-liver chimeric uPA^+/+^-SCID mice (hepatocyte donor C342) or with naïve serum/phosphate-buffered saline (control mice, black dots) 1 day prior to HBV/HDV co-infection (week 0) (B–C). Blue triangles (n=2): adoptive transfer of antisera obtained from D7/D8 protein mix vaccinated mice. Red triangles (n=3): adoptive transfer of antisera obtained from D4 DNA prime-D7/D8 protein mix boost group. Blood plasma was collected at week 4, 6, 8, 12, 16 and time point of sacrification (week 20). HBV DNA (B) and HDV RNA (C) levels measured in mouse plasma. Limit of detection (LOD) for HBV at 1:10 dilution is 3.750 IU/mL; LOD for HDV at 1:10 dilution is 187.5 IU/mL. PreS1A (D) and preS1B (E) antibody titres were determined in mouse plasma via ELISA as end point serum dilutions (at logarithmic scale) at which the optical density (OD) value at 405 nm was at least twice the OD of the negative control (naïve mouse serum) at the same dilution. The cut-off was set at 60.

Immunised mice received either (i) D4 DNA prime-D7 protein boost, (ii) D4 DNA prime-D8 protein boost, (iii) D7-D7 protein prime-boost or (iv) D8-D8 protein prime-boost, which resulted in comparably high anti-preS1 IgG titres (10^4^–10^5^ on a log scale) (figures 2A and 4A). Therefore, we passively immunised human-liver chimeric uPA^+/+^-SCID mice with either a pool from (i) with (ii), n=3; or (iii) with (iv), n=2; or naïve mouse serum/PBS to assess potential prevention of HBV/HDV co-infection (figure 4 and online supplemental table 1). One day later, all mice were challenged with HBV/HDV (5×10^6^ IU HBV/mouse and 1.48×10^6^ IU HDV/mouse, both cell-culture derived). All control mice (n=6) acquired HBV (figure 4B) and HDV (figure 4C) infection with rapidly increasing plasma titres from week 4 postinfection onwards with HBV DNA and HDV RNA levels of around (or at least) 10^7^ IU/mL at the end of the study (week 20 postinfection). Remarkably, all mice passively immunised with either of the two antiserum regimens were completely protected from both HBV and HDV at all assessed time points (figure 4B, C). Evaluation of preS1-antibody titres in mouse plasma 1 day after a single injection of antiserum shows end point serum dilutions between 10^3^ and 10^4^ (on a log scale) and decline to undetectable levels 2 weeks later (figure 4D, E). One control mouse unexpectedly tested positive at week 4 for preS1A antibody. This was likely caused by contamination, but the sample could not be retested because of limited volume.

### Active immunisation with preS1-HDAg induces preS1-antibodies and HDV-specific T-cell responses in a model of the HBsAg chronic carrier

We have seen so far that an effective vaccine able to induce broadly reactive HBV/HDV-specific T-cells and neutralising antibodies to both HBV and HDV should ideally contain both HDV gt1-2 and preS1 sequences based on a heterologous DNA prime-protein boost approach (figure 2 and online supplemental figure 3). Therefore, we synthesised a fusion protein construct containing the combination of D7 and D8 sequences linked to preS1 (online supplemental table 1) and used this vaccination scheme (D4 DNA prime and D7-8 fusion protein boost) to test if it can circumvent the HBsAg-induced tolerance present in the chronic HBV-infected host.^39^

**Figure 5:**
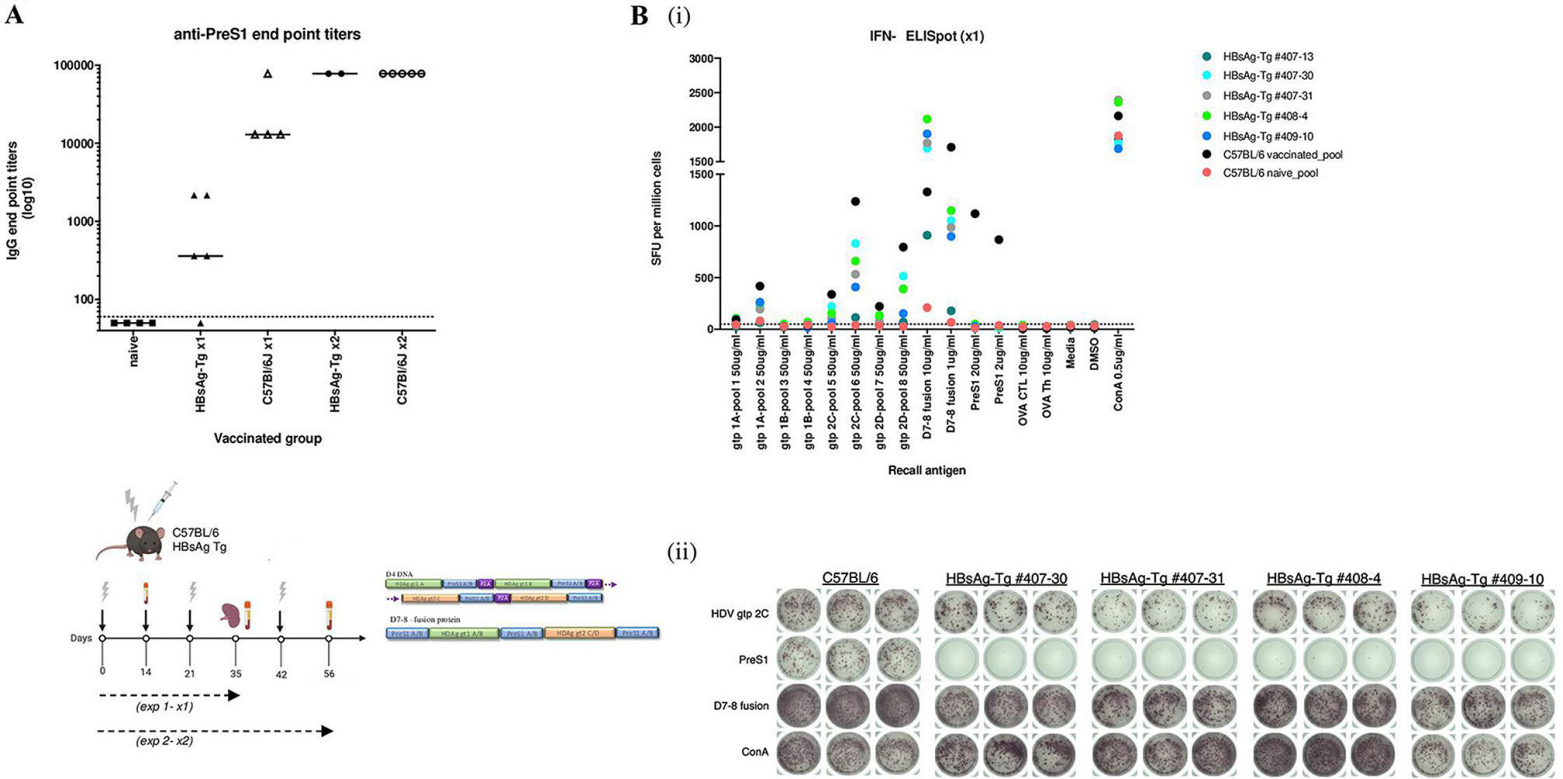
Immunogenicity of heterologous prime-boost vaccination in the HBV chronic carrier. Hepatitis B surface antigens (HBsAg)-transgenic (Tg) and wild-type C57BL/6 mice were immunised with D4 DNA and boosted once (‘x1) or twice (‘x2) with D7-D8 fusion protein construct in two independent experiments. Two weeks after last boost, blood and splenocytes from each vaccinated mouse were collected for subsequent ELISA (A) and interferon (IFN)-γ enzyme-linked immunospot assay (ELISpot) (B) assays, respectively. (A) Mean anti-preS1 titres for each vaccinated group were determined as end-point serum dilutions (at logarithmic scale) at which the optical density (OD) value at 405 nm was at least twice the OD of the negative control (naïve mouse serum) at the same dilution. The cut-off was set at 60. Each symbol represents one individual mouse. (B) Number of IFN-γ secreting T-cells after 48 hours stimulation with preS1 and HDV antigens for individual HBV-Tg mice or pooled C57BL/6 mice (n=4) after one boost (x1). (Bi) Dot plots summarise the frequencies of IFN-γ-producing cells and individual dots represent the mean number of spot-forming cells per million from triplicate wells tested for each antigen condition for individual HBV-Tg mice or C57BL/6 pools. (Bii) Representative ELISpot images of IFN-γ spot-forming cells for C57BL/6 vaccinated group and HBV-Tg mice showing responses to HDV gt2C peptide pool, preS1 peptide, D7-8 fusion protein antigen and ConA. HDV peptide pools 1–4 correspond to gt1 and pools 5–8 to HDAg gt2. Pools 1 and 2 of gt1 refer to sequence/isolate A, while pools 3 and 4 correspond to sequence/isolate B. Similarly for HDV gt2, pools 5 and 6 refer to sequence/isolate C and pools 7 and 8 to sequence/isolate D. Each pool contained 20 or 21 (for pools 1 and 5) 15-mer peptides with 10 amino acids (aa) overlap. PreS1 peptide refer to the consensus sequence (total 4 7aa; region 2–48 aa), while cytotoxic T-lymphocyte and T-helper ovalbumin peptides (respectively OVA CTL and OVA Th) at 10 µg/mL were used as negative controls. Concanavalin A (ConA) was used as assay positive control at 0.5 µg/mL. Image was created with BioRender.com.

The HBsAg-Tg mouse model used in this study expresses the MT-PSX transgene and has zinc-inducible mouse metallothionein-I promoter sequences which direct expression of the L-HBsAg protein, while expression of S-HBsAg and M-HBsAg envelope proteins are regulated by a constitutively active internal promoter located within the envelope coding region on the transgene. Therefore, in absence of dietary zinc supplementation, S-HBsAg and M-HBsAg proteins, but not L-HBsAg envelope protein, are expressed, resulting in high serum levels of HBsAg (based on HBsAg ELISA, *data not shown*) with very low hepatocellular retention, similar to the human infection. Importantly, on vaccination of these mice after one boost (figure 5A, ‘x1’), preS1-antibodies were induced, although much lower when compared with the C57BL/6 vaccinated group (titres around 10^3^ vs 10^5^, respectively). However, two protein boosts in a second independent experiment (figure 5A, ‘x2’), resulted in antibody levels comparable to vaccinated C57BL/6 wild-type mice (titres at 10^5^). Interestingly, this vaccination approach also elicited high HDV-specific T-cell responses for both genotypes, but no preS1-directed IFN-γ secreting T-cells in HBsAg-Tg mice following one boost, in contrast to the observations in C57BL/6 vaccinated mice (figure 5Bi, ii). This confirms the T-cell tolerance to HBsAg-derived sequences in the transgenic mouse model, and that our immunotherapy can effectively circumvent this T-cell deficiency based on induction of preS1-antibodies and HDV-specific T-cells. One mouse (HBsAg-Tg #407–13) did not develop preS1-antibodies (figure 5A), but was also lacking HBV/HDV-specific T-cells after individual assessment (figure 5Bi). The response observed after in vitro stimulation with D7-D8 fusion protein (figure 5Bi) can be most probably attributed mainly to the HDV component of the protein.

### Pres1-HDAg antisera from vaccinated mice (partly) protect HBV-infected mice from HDV superinfection

**Figure 6:**
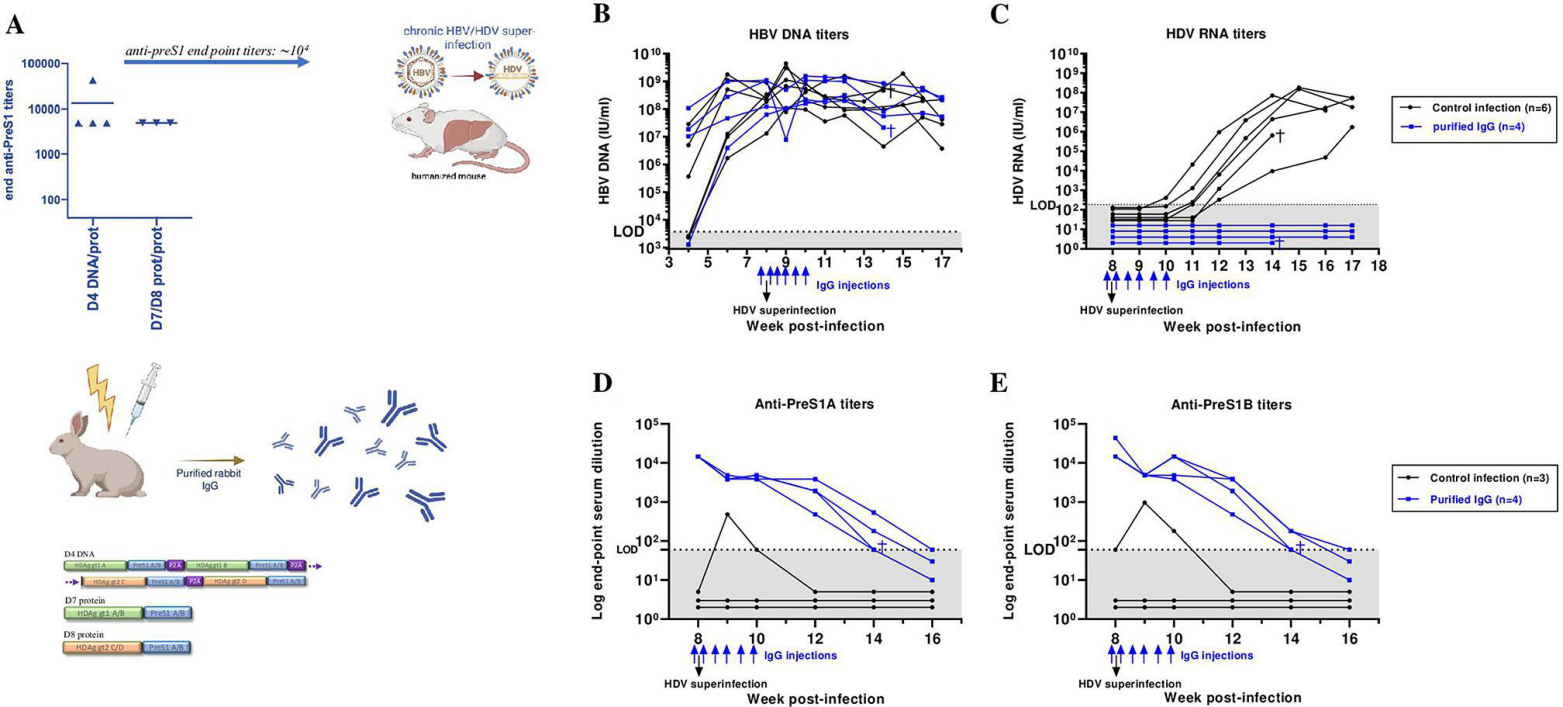
Prevention of HDV superinfection in vivo. (A) New Zealand white rabbits were immunised with DNA/protein preS1-HDAg harbouring regimens and induction of preS1-antibodies was assessed by ELISA. Anti-preS1 end point serum dilutions are shown at logarithmic scale. IgGs from rabbit antiserum was purified and pooled based on comparable antibody titres for use in this in vivo superinfection setting. (B–E) Six human-liver chimeric uPA^+/+^-SCID mice (hepatocyte donor C342) were infected with cell-culture or patient-derived HBV at week 0 and accordingly superinfected with HDV at week 8. One day prior to superinfection, 3/6 cell-culture inoculum infected and 1/4 patient inoculum infected mice intrasplenically received 100 µL purified rabbit preS1-IgGs. In addition, these mice received repeated IgG injections (of 100 µL) intraperitoneally at day 1, 4, 7, 11 and 14 post-HDV superinfection. One mouse received the same dosing schedule, but with naïve serum, the other control mice were non-treated (black dots). Blood plasma was collected at week 4, 6, 8, 9, 10, 11, 12, 14, 16 and end point (week 17). HBV DNA (B) and HDV RNA (C) titres in mouse plasma. Limit of detection (LOD) for HBV at 1:10 dilution is 3.750 IU/ml; LOD for HDV at 1:10 dilution is 187.5 IU/mL. (D–E) PreS1A and preS1B antibody titres, respectively, represented as end point serum dilutions (at logarithmic scale) at which the optical density (OD) value at 405 nm was at least twice the OD of the negative controls at the same dilution. The cut-off is set at 60. Image was created with BioRender.com.

In order to evaluate the ability of our vaccine strategy to induce antibody responses in a larger animal species and at the same time obtain higher amounts of sera and thus purified IgG, we immunised rabbits with D4 DNA prime and boosted them with D7/D8 protein mix in adjuvant, or primed and boosted with the D7/D8 protein mix in adjuvant (figure 6A). Both groups showed similar antibody -titres reaching at least 10^4^ and therefore anti-sera were pooled and IgG-purified (figure 6A). Next, we investigated if passive immunisations with these preS1-antibodies could mediate protection against HDV superinfection in humanised mice chronically infected with HBV. Therefore, 10 mice were initially inoculated with HBV and showed increasing HBV DNA levels in plasma until a plateau was reached at week 8 (figure 6B). Then, six mice were superinfected with cell-culture-derived HDV and four mice with patient-derived HDV, of which respectively three and one mice were passively immunised with purified vaccine-induced IgG (5.5 mg/mouse, 100 µL intrasplenically) 1 day prior viral challenge. The same IgG dose was administered intraperitoneally at day 1, 4, 7, 11 and 14 post-HDV superinfection. The remaining mice received either the same dosing schedule with naïve IgG (n=1) or no treatment (n=5). These controls show detectable HDV RNA levels in plasma already 3 weeks following superinfection, reaching around 2×10^7^ IU/mL at week 6 post-HDV superinfection (figure 6C). Although no effect was observed on HBV DNA levels, most probably due to the already established and widely spread HBV infection (figure 6B), we show that all four passively immunised were completely protected from acquiring an HDV superinfection (figure 6C). We also show high preS1A and preS1B antibody titres between 10^3^ and 10^5^ that become undetectable 2–4 weeks following the final antiserum injection (figure 6D–E). We note that two samples from a control treated mouse (receiving naïve serum) scored slightly positive for the presence of preS1-specific antibodies, most likely caused by an unspecific reaction. As a confirmation, we also included mice transplanted with hepatocytes from another hepatocyte donor (L191501; online supplemental figure 4). Here, six humanised mice were initially infected with patient-derived HBV (same dosing as in previous set-up) and 1 day prior to superinfection with patient-derived HDV (8 weeks after HBV infection), four out of six mice were passively immunised with 100 µL total antiserum derived from mice vaccinated with preS1-HDAg D7/D8 protein mix in QS21 adjuvant (online supplemental figure 4), while two mice were treated with naïve mouse serum (online supplemental figure 4B, C). Antiserum injections (50 µL) were additionally given intraperitoneally at day 1, 4 and 7 postsuperinfection. Control mice established detectable HDV titres 1 week after superinfection (online supplemental figure 4C), reaching peak levels around week 12 and all mice harboured increasingly positive HBV DNA titres in plasma (online supplemental figure 4B). Interestingly, we clearly noted attenuated infection for the antiserum-treated mice: two mice show delayed HDV viraemia (at week 12 and at week 14), while the other two repetitively tested negative until at least week 10 and week w12, after which they spontaneously died (online supplemental figure 4C). Also, preS1A and preS1B ELISA showed titres around or above 10^4^ that became negative 3 weeks following the final injection (online supplemental figure 4D, E).

## Discussion

Current antiviral therapies for chronic HBV infection can suppress viral load and slow down disease progression, but unfortunately cannot always eliminate the virus or prevent liver disease and cancer.^44^ Multiple new antivirals are in the pipeline, but it is widely accepted that host immune stimulation is required to circumvent the immune evasion strategies used by HBV for off-therapy control.^34 45^ This could lead to the highly desired functional cure, that is, stable loss of circulating HBsAg, especially when targeting immune tolerant chronic carriers.^33 46 47^ Moreover, eliminating HBV directly implies eradicating HDV, which is considered as the most harmful type of viral hepatitis and for which no specific antivirals are available.^3 13^

Previously, we designed an immunotherapy approach based on preS1 sequences to induce HBV/HDV-specific entry-inhibiting antibodies that are additionally linked to HDAg, acting as an epitope to prime naïve T-cells towards both preS1 and HDAg.^35^ We hypothesised that this may bypass the induced T-cell tolerance in the chronic HBsAg carrier. Since we have previously shown effective HBV neutralisation in vitro and partial protection of human-liver chimeric mice from HBV mono-infection, we now optimised our vaccination approach and extended our evaluation towards prevention of HDV as well.

Throughout this study, various vaccination approaches were evaluated to determine the most immunogenic strategy while at the same time considering aspects of clinical applicability including construct design and manufacturing, boost doses and protein adjuvants (figure 1 and online supplemental table 1). As such, the heterologous D4 DNA prime-protein boost approach was superior in inducing both high preS1-antibody responses (figure 2A) and HBV/HDV-specific T-cells (figure 2Bii, iii and online supplemental figure 3) compared with the homologous D7 and D8 protein approach that could also elicit comparable antibody levels (figure 2A), but much lower HBV/HDV T-cell responses (figure 2Bi, iv, v). Of note, although different schemes evoked slightly different responses mainly with regard to T-cells, all in vitro and in vivo neutralisation experiments were conducted using comparable antibody titres (serum end point dilutions of around 10^4^–10^5^ at logarithmic scale) (figures 3A, 4A and 6A).

Using appropriate in vitro systems,^48 49^ we show that transfer of induced preS1-antibodies effectively neutralised both HBV and HDV in vitro as prevention achieved with MyrB (figure 3B, C), a synthetic preS1 peptide-based competitive inhibitor for the HBV/HDV receptor NTCP.^50^ However, this inhibitor has been shown to cause alterations in bile acid transport and metabolite functions of NTCP and its long-term daily subcutaneous administration to reach optimal antiviral activity for patients with chronic HDV is rather inconvenient.^50–52^ Importantly, our strategy implies antiserum harbouring preS1-antibodies that target both viruses directly instead of the receptor, thereby not disturbing the natural function of this host protein.

Next to protecting mice from HBV mono-infection, as was shown in our previous work,^35^ we demonstrate here that the improved immunotherapy evoked complete protection against HBV/HDV co-infection in vivo during a 20-week follow-up period (figure 4).

Moreover, we investigated if active heterologous immunisation could also elicit adaptive immune responses in HBsAg-Tg mice, representing a model of tolerance in the chronic HBsAg carrier.^39^ We show that our preS1-HDAg approach was able to induce preS1-titres of 10^3^ end point serum dilution at log scale after one booster dose (figure 5A; x1), which increased to titres at 10^5^ comparably to the vaccinated C57BL/6 mice following two booster doses (figure 5A; x2). Although HDV T-cell responses were induced after recall with the respective HDAg-antigens in all vaccinated groups (with exception of one HBsAg-Tg mouse that did not develop any T-cells or preS1-antibodies), very weak preS1-specific T-cells could be observed in the HBsAg-Tg mice compared with the wild-type (figure 5Bi, ii) after one booster vaccine dose. This confirms the T-cell tolerance to HBsAg-derived sequences in these mice, and that our immunotherapy can effectively circumvent this T-cell deficiency through induction of preS1-antibodies and HDV-specific T-cells.

Interestingly, other HBV vaccine studies support the role of preS1 alone to overcome tolerance in the chronic carrier,^37^ however in our case we saw that (at least) one preS1-HDAg vaccine boost was not able to elicit sufficiently preS1-specific T-cell responses. Overall, only HDAg-induced T-cells and importantly preS1-antibodies were induced, confirming the importance of HDAg inclusion to prime naïve T-cells and support endogenous antibody production, even in this setting of HBsAg-induced tolerance in the chronically infected host.

Importantly, we further demonstrate protection of HBV-infected humanised mice from acquiring HDV superinfection (figure 6) on transfer of immunotherapy-induced preS1-antibodies. Although no therapeutic effect was observed on HBV, most likely due to the already established high levels and spread of infection (figure 6B), HDV RNA levels remained undetectable in all inoculated mice (figure 6C). In mice repopulated with hepatocytes from a different donor, at least partial prevention (either complete protection or delayed kinetics) of HDV superinfection could be achieved (online supplemental figure 4). Important to note is that hepatocytes from this donor (L191501) seemed to be much more susceptible for infection (*data not shown*), which could explain why prevention was not entirely achieved. In addition, there are experimental differences regarding type of immunoglobulin preparation used (50 µL total murine antiserum vs 100 µL purified rabbit IgGs) and dosing schedule (4 vs 6 injections) (online supplemental figure 4 and figure 6, respectively).

Notably, although the commercially available HBV vaccines also render protection against HDV in healthy people, chronic HBV patients do not respond to traditional HBV vaccination due to their tolerized immune status towards S-HBsAg.^53^ Consequently, the impact of our HDV superinfection prevention experiment is highly valuable because this strategy would provide protection to patients with chronic HBV from acquiring a harmful HDV superinfection.

Different strategies to establish a protective HDV vaccine have been investigated in the woodchuck model^14 15^: synthetic peptides derived from B-cell epitopes of HDAg^16^; p24-HDAg expressed in *E. coli*,^17^ yeast^19^ and baculovirus^18^; vaccinia virus expressing p24/p27-HDAg^18 20^ and finally, DNA vaccination with vectors expressing p24-HDAg.^21^ However, although protein immunisation induced specific antibody responses, no protection against HDV superinfection could be achieved. Using the vaccinia virus and DNA immunisation approach, only modulation of the course of HDV superinfection could be seen in certain woodchucks, but no protective immune response. Research on HDAg-DNA vaccination confirmed that both a cellular immune response and HDAg-specific antibodies can be induced in mice.^23 24^ Furthermore, a DNA prime/adenoviral boost immunisation protocol was able to prevent WHV-HDV co-infection in woodchucks.^22^ To our knowledge, no prevention of HDV superinfection has yet been shown in woodchucks, or any other models.

In conclusion, we have developed a vaccine immunotherapy that uses HDAg as a heterologous antigen to circumvent HBsAg-specific T-cell tolerance while simultaneously promoting preS1-antibody production in a setting of chronic infection. These antibodies effectively prevent HBV/HDV co-infection and HDV superinfection in vivo. As this therapy targets both the entry step and the translation of viral proteins, it can efficiently complement existing therapies that block viral maturation. In addition, it is likely that preS1-antibodies targeting HBV and HDV virions, shuttle these virions to antigen-presenting cells thereby triggering additional priming of T-cell responses to HBsAg, HBcAg and the polymerase. This may further assist the host immune system to control HBV and/or HDV infection. Our immunisation approach provides an interesting and overall safe step towards achieving a functional cure.

## Data Availability

All data relevant to the study are included in the article or uploaded as supplementary information.
