# Supplementary material for: Novel prime-boost immune-based therapy inhibiting both hepatitis B and D virus infections
Source: Gut. 2022 Aug 17;72(6):1186–95. doi: 10.1136/gutjnl-2022-327216 (PMC10176361; doi:10.1136/gutjnl-2022-327216)
Supplement: Supplementary data [file gutjnl-2022-327216supp001.pdf]

SUPPLEMENTAL MATERIAL AND METHODS

Animals

Female C57BL/6J (H-2b) mice were obtained from Charles River Laboratories (Sulzfeld, Germany) and maintained under standard conditions at Karolinska Institutet (Karolinska University Hospital Huddinge, Preclinical Laboratory (PKL), Sweden). B6;SJL-Tg(Mt1-HBV)28Bri/ChiJ (HBsAg) mice were purchased as embryos from JAX® laboratories. All mice were 8-10 weeks old at the start of immunizations. New Zealand White rabbits were purchased from commercial vendors and kept at Astrid Fagreus Facility/Comparative Medicine Fagreus (KMF) at Karolinska Institutet. Human-liver chimeric uPA$^{+/+}$-SCID mice were produced as previously described and maintained at the central animal facility of the Faculty of Medicine and Health Sciences of Ghent University (Ghent, Belgium) under standard conditions [42, 43]. All chimeric mice were 12-14 weeks old at the start of infections. All animal procedures were approved by the local Animal Ethical Committees in Sweden and Belgium.

Synthesis and evaluation of preS1/HDAg fusion vaccine constructs

All fusion constructs D4, D7 and D8 harbor different combinations of L-HDAg (genotype 1 and 2) and preS1A/B consensus sequences (aa 2-48) **(Fig. 1)**. The HDAg sequences were obtained from four different clinical isolates: US-2 and CB; and 7/18/83 and TW2476. D4 DNA construct generation and evaluation were conducted exactly as previously described [35]. Briefly, gene was cloned into the pVAX1 backbone (Invitrogen, Carlsbad, CA) using restriction sites EcoRI and HindIII. Plasmid was grown in TOP10 *E. coli* cells (Life Technologies, Carlsbad, CA) and purified for *in vivo* injections using Qiagen Endofree DNA purification kit (Qiagen GmbH, Hilden, Germany) following manufacturer´s instructions. The correct gene size was confirmed by restriction enzyme digests using EcoRI and HindIII (Fast Digest, Thermo Fisher Scientific). For the three recombinant proteins, namely D7, D8 and D7-D8 fusion protein constructs, the vector pET-30a(+) with His-tag (C-terminal) was used with the *E. coli* BL21 Star (DE3) expression system. After refolding, D7 and D8 protein purification was conducted by one-step Ni column, purification of D7-D8-fusion protein following three-step purification, i.e. Ni

column, SP Sepharose column and Superdex 200 column. All produced protein constructs have passed QC control and were assessed for endotoxin removal and purity. Endotoxin levels were quantified as 2,6EU/mg (D7 protein), 0,73EU/mg (D8 protein) and below 100 EU/mg (D7-D8 fusion protein construct). Purity was 85% for both D7 and D8 protein constructs and 65% (and 85% in second batch) for the D7-D8 fusion construct. Synthesis, sub-cloning, upscale production, protein purification and expression analysis were all conducted by GenScript (**Suppl. Fig. 1**).

Immunization protocols in mice and rabbits for preS1/HDAg antiserum production

Wild-type C57BL/6J or HBsAg-Tg mice were immunized with D4 DNA vaccine construct and boosted once or twice with recombinant protein constructs D7 and D8, either individually, as mix, or as fusion protein construct. Mice were sacrificed two weeks after the last vaccination for spleen and blood collection as previously described [35]. In detail, female C57BL/6J mice (five per group) were immunized intramuscularly (i.m.) in the *tibialis cranialis anterior* (TA) muscle with 50 µg plasmid DNA in a volume of 50 µL (in sterile PBS) by regular needle (27G) injection, followed by *in vivo* electroporation using the Cliniporator device (IGEA, Carpi, Italy). During *in vivo* electroporation, a 1 ms 600 V/cm pulse and an additional 400 ms 60 V/cm pulse pattern was employed to facilitate appropriate uptake of the plasmid. For vaccinations with recombinant proteins, 10 µg for each individual D7 or D8 protein, or 20 µg of a D7/D8 mixture or fusion construct were administered subcutaneously at the mouse tail base in a total injectable volume of 100 µl in respective protein adjuvants (**Suppl. Table 1**). Prior to identifying QS21 adjuvant as a more immunogenic adjuvant (**Fig. 3A**), alum was used as protein adjuvant in some of the animal vaccine studies (**Fig. 6** and **Suppl. Table 1**). QS21 is a purified plant extract derived from the Soap bark tree (*Quillaja saponaria*). The extract contains water soluble triterpene glucoside compounds, which are members of a family of plant-based compounds called saponins. Mice were given analgesics prior to vaccinations and kept under isoflurane anesthesia during all vaccination procedures. For immunizations in rabbits, four New Zealand White rabbits per group were immunized with 300 µg of D4 DNA vaccine construct by i.m. injection in 300 µL sterile PBS to the right TA muscle followed by *in vivo* electroporation as has previously been described [35]. Protein

constructs were administered i.m. at a dose of 100 µg in Imject Alum adjuvant. Detailed information for each *in vivo* experimental set-up is also given in **Suppl. table 1**.

Evaluation of prime-boost immunogenicity by enzyme-linked immunosorbent assay (ELISA) & enzyme-linked immunospot assay (ELISpot)

In order to determine the antibody titers of vaccinated mice and rabbits, ELISA was performed exactly as has previously been described following an in-house established protocol [35, 40]. Briefly, mouse and rabbit sera were assessed for reactivity against two consensus peptides (10 µg/ml) of the preS1 domain of HBsAg (preS1A and preS1B) consisting of 47 aa and purchased from Sigma-Aldrich (St. Louis, MO). The peptides have passed QC (Sigma-Aldrich PEPscreen® Directory) and have >70% purity. Antibody titers were determined as end-point serum dilutions at which the OD value at wavelength 405 nm is at least twice the OD of the negative controls (non-immunized or control mouse serum) at the same dilution.

Induction of HBV and HDV specific T-cells after vaccinations was evaluated by analyzing IFNγ secretion of harvested splenocytes using ELISpot as has previously been described [35]. Briefly, two weeks after final vaccination, splenocytes from each vaccinated group were pooled and (re)stimulated *in vitro* with pools of peptides corresponding to the L-HDAg of gt1 and gt2 and the two individual consensus preS1 peptides. Extended information on the peptides are given in **Suppl. Table 2**. IFNγ secretion was evaluated 48h later according to instructions of a commercially available ELISpot assay (Mabtech, Nacka Strand, Sweden).

HBV/HDV viral inocula

HBV/HDV viral inocula were generated for *in vitro* and *in vivo* neutralization assays. HBV (gtD) was produced using the tetracycline-inducible cell line HepAD38 [41]. Supernatant was collected after removal of tetracycline, concentrated using PEG-it™ Virus Precipitation Solution 5x (System

Biosciences), aliquoted and stored at -80°C. For HDV (gt1) production, plasmids pSVL(D3) and pT7HB2.7 were co-transfected using TransIT-LT1 transfection reagent (Mirus Bio) in Huh7.5 cells and supernatant was collected, concentrated and stored exactly the same way as for HBV. Plasma from HBV mono- and HBV/HDV co-infected patients was used as viral inoculum in the *in vivo* neutralization experiments and as a source of primary antibody for HDV-specific immunofluorescent stainings.

***In vitro* neutralization of HBV and HDV with mouse preS1/HDAg antiserum**

For prevention of both HBV and HDV infection *in vitro*, HepG2.hNTCP cells were seeded (10.000 cells/well in 100 µl) in Poly-L-Lysine coated 96-well plates in Dulbecco's Modified Eagle's Medium (DMEM) supplemented with 10% fetal calf serum, 1% non-essential amino acids, 1% L-glutamine and antibiotics (referred to as 'complete DMEM'). Three days later, the HBV inoculum (2.530 IU/cell) or HDV inoculum (4 IU/cell) in complete DMEM supplemented with 2,5% DMSO and 4% PEG8000 (referred to as 'infection medium') was incubated for 1h at room temperature with different dilutions of D7/D8 protein prime/boost mouse antiserum with QS21 adjuvant (**Suppl. Table 1**): 1/100 dilution and then 1:3 serial dilutions until 1:72.900. Simultaneously, naïve serum was included and all conditions were performed in duplicate. This virus-antiserum mixture was then added to the HepG2.hNTCP cells (200 µl) and incubated for 24h. As positive control for prevention of infection, myrcludex B (myrB) was applied to the cells at 200 nM and 1h prior to virus incubation. After 24 hours, cells were washed three times with PBS to remove viral inoculum and every 2 to 3 days, the antiserum/naïve mouse serum or myrB was refreshed in infection medium (but without 4% PEG8000) until read-out 8 days following infection by immunofluorescent (IF) staining.

HBV and HDV IF staining and imaging

For HBcAg staining, cells were fixed (4% paraformaldehyde, 20 min), permeabilized (1% Nonidet® P40-Substitute, 15 min) and blocked (PBS + 2% BSA + 10% goat serum) overnight at 4°C. Cells were stained with a polyclonal rabbit HBc-antibody (11 µg/ml) for 1 hour (DakoCytomation, REF-B0586),

followed by 1 hour incubation of an AlexaFluor-488 labeled goat anti-rabbit IgG secondary antibody at 2 µg/ml (Invitrogen, REF-A11034). HDV IF staining was similarly conducted with some slight modifications to the above-described procedure: blocking was performed with 5% skim milk in PBS, HDV patient EDTA-plasma was used at a 1/2000 dilution as primary antibody, followed by an AlexaFluor-488 goat anti-human IgG secondary antibody at 2 µg/ml (Invitrogen, REF-A11013). Nuclei were stained using DAPI at 1,43 µg/ml (MolProbes, REF-D3571). Imaging was performed using the Leica TCS-SPE confocal microscope (20X objective). Per well, 3 random pictures were taken and all conditions were performed in duplicate (6 random pictures per condition). Automated cell counting was performed using ImageJ software v1.53c.

Generation of human-liver chimeric uPA$^{+/+}$-SCID mice and passive immunizations

Approximately 10^6 primary human hepatocytes (donor C342 from Corning, The Netherlands or donor L191501 from Lonza, Switzerland) were transplanted into homozygous uPA$^{+/+}$-SCID mice as previously described [42, 43]. Human albumin levels in mouse plasma, determined by ELISA (Bethyl Laboratories, Montgomery, Texas, USA), were used as marker to assess successful liver humanization.

For passive immunizations, isoflurane-anesthetized human-liver chimeric uPA$^{+/+}$-SCID mice were intrasplenically (i.s.) injected with 100 µl total mouse/rabbit antiserum or 5,5 mg purified rabbit IgG (Pierce Protein A kit, Thermo Fisher Scientific) one day prior to HBV/HDV co-infection or HDV superinfection. Additional purified IgG injections (100 µl-5,5 mg) were intraperitoneally (i.p.) administered at day 1, 4, 7, 11 and 14 following HDV superinfection in hepatocyte donor C342 transplanted mice (**Fig. 6**) and additional total mouse antiserum (50 µl) in hepatocyte donor L191501 transplanted mice at day 1, 4 and 7 following HDV superinfection (**Suppl. Fig. 4**). Control mice were either injected with naïve serum or PBS. In **Suppl. Table 1**, a detailed overview of all experimental set-ups is shown. For HBV/HDV co-infections, mice were injected i.p. with 100 µl of viral inoculum consisting of $5x10^6$ IU HBV and $1,48x10^6$ IU HDV (both cell-culture derived). For HDV superinfections, mice were first infected with HBV ($5x10^6$ IU-cell culture derived, or 10^6 IU patient-

derived HBV) and 8 weeks later with HDV (2,55x10^5 IU cell culture or patient-derived HDV). Blood plasma was collected and the HBV/HDV viral load in mouse plasma was determined by RealStar® HDV/HBV (RT-) qPCR (Altona Diagnostics, Germany) following total nucleic acid extraction (NucliSENS® EasyMag®, BioMérieux, France). Also the preS1-titer in blood plasma was determined by ELISA following passive immunization as described above. Mice were sacrificed at week 20 post co-infection or at week 9 post-superinfection. All animal procedures were approved by the local Animal Ethical Committees in Sweden and Belgium.

Patients and public involvement

Patients or the public were not involved in the design, or conduct, or reporting, or dissemination plans of our research.

Statistical analysis

Data was analyzed using GraphPad Prism V8 software.

SUPPLEMENTAL TABLES AND SUPPLEMENTAL FIGURE LEGENDS

Suppl. Table 1: Summary of different vaccination strategies used in all *in vitro* and *in vivo* neutralization studies.

<table>
<tr><th>Experiment</th><th>Immunized species</th><th>Vaccinated groups</th><th colspan="2">Adjuvants</th><th>Number of boosts</th><th>ELISA</th><th>Antibody titers</th><th>ELISpot</th><th>Sera used for in vitro experiment</th><th>Sera used for in vivo experiment</th></tr>
<tr><td>Prevention of HBV and HDV infection in vitro (Fig. 3)</td><td>Mouse</td><td>D7/D8 protein mix</td><td colspan="2">Alum, QS21,MF59</td><td>1x</td><td>+</td><td>$\sim10^5$ (for QS21)</td><td>+</td><td>D7/D8 protein mix in QS21</td><td>N/A</td></tr>
<tr><td rowspan="4">Prevention of HBV/HDV co-infection in vivo (Fig. 4)</td><td rowspan="4">Mouse</td><td>i. D4 DNA prime & D7 boost</td><td>DNA: EP</td><td>Protein: IFA</td><td rowspan="4">2x</td><td rowspan="4">+</td><td rowspan="4">10^4 - 10^5</td><td rowspan="4">+</td><td rowspan="4">N/A</td><td rowspan="4">Pooled i & ii (group 1) and pooled iii & iv (group 2)</td></tr>
<tr><td>ii. D4 DNA prime & D8 boost</td><td>DNA: EP</td><td>Protein: IFA</td></tr>
<tr><td>iii. D7-D7 prime-boost</td><td colspan="2">IFA</td></tr>
<tr><td>iv. D8-D8 prime-boost</td><td colspan="2">IFA</td></tr>
<tr><td rowspan="2">Prevention of HDV superinfection in vivo - exp1 (Fig. 6)</td><td rowspan="2">Rabbit</td><td>i. D4 DNA prime & D7/D8 protein mix boost</td><td>DNA: EP</td><td>Protein: Alum</td><td rowspan="2">2x</td><td rowspan="2">+</td><td rowspan="2">$\sim10^4$</td><td rowspan="2">N/A</td><td rowspan="2">N/A</td><td rowspan="2">Pooled i & ii, purified IgGs</td></tr>
<tr><td>ii. D7/D8 protein mix boost</td><td colspan="2">Alum</td></tr>
<tr><td>Prevention of HDV superinfection in vivo - exp2 (Suppl. Fig. 4)</td><td>Mouse</td><td>D7/D8 protein mix</td><td colspan="2">Alum, QS21,MF59</td><td>1x</td><td>+</td><td>$\sim10^5$ (for QS21)</td><td>+</td><td>N/A</td><td>D7/D8 protein mix in QS21</td></tr>
</table>

N/A: not assessed.

Suppl. Table 2. Overlapping HDV peptide pools used in the ELISpot experiments.

Peptide pools	Peptides	Sequence	Isolate	Genotype
Pool 1	1-21	A	US-2	1
Pool 2	22-42	A	US-2	1
Pool 3	43-63	B	CB	1
Pool 4	64-84	B	CB	1
Pool 5	1-21	C	7_18_83	2
Pool 6	22-42	C	7_18_83	2
Pool 7	43-63	D	TW2476	2
Pool 8	64-84	D	TW2476	2

The HDAg sequences were obtained from four different clinical isolates: US-2 and CB; and 7/18/83 and TW2476.

Suppl. Figure 1. Characterization of protein constructs by SDS-PAGE and Western blot. All protein constructs express expected sizes; **(A)** D7 protein length: 527 aa, MW: 59,5 kDa, **(B)** D8 protein length: 527 aa, MW: 59,2 kDa and **(C)** D7-D8 fusion protein length: 1145 aa, MW: 128,6 kDa. **(A-C)** M1 and M2 refer to protein markers, lane 1 to BSA control (2,00 µg) and lanes 2 and 3 to D7, D8 and D7-D8 fusion constructs respectively, under reduced conditions. As primary antibody, a commercially available mouse-anti-His mAb was used. Data was provided by GenScript.

Suppl. Figure 2. ELISA (A) and HBV (B) *in vitro* neutralization for all mice groups vaccinated with different combinations of DNA/protein preS1-HDAg or only HDAg constructs. **(A)** Mean anti-preS1 end-titers at logarithmic scale per vaccinated group in which each dot represents one individual

mouse. Antibody titers were determined in mouse serum via ELISA as end-point serum dilutions at which the OD value at 405nm was at least twice the OD of the negative control (naïve mouse serum) at the same dilution. The cut-off was set at 60. **(B)** The induced preS1-antibodies from each vaccinated group were also assessed for their ability to neutralize HBV *in vitro*. Graph shows relative infection based on HBeAg ELISA for each regimen concentration at logarithmic scale. Monoclonal antibody Ma18/7 was used as positive control and naïve serum as negative control.

Suppl. Figure 3. Immunogenicity of heterologous prime-boost strategy based on D7-D8 fusion protein construct in C57BL/6 mice.

Mice primed with D4 DNA and boosted twice with D7-D8 fusion protein construct in QS21 adjuvant. Two weeks after the final boost, splenocytes were collected and pooled for subsequent ELISpot assay. Induction of HBV/HDV-specific T-cells after 48h stimulation with indicated HBV and HDV antigens. Bars represent the mean number of IFN-γ spot-forming cells (SFCs) per million with standard error from triplicate wells tested for each antigen condition. The different bar colors correspond to the respective sequences of the vaccine constructs, i.e. green for HDAg gt1, orange for HDAg gt2 and blue for preS1 A/B. HDV peptide pools 1-4 correspond to gt1 and pools 5-8 to HDAg gt2. Pools 1 and 2 of gt1 refer to sequence/isolate A, while pools 3 and 4 correspond to sequence/isolate B. Similarly for HDV gt2, pools 5 and 6 refer to sequence/isolate C and pools 7 and 8 to sequence/isolate D. Each pool contains 20 or 21 (for pools 1 and 5) 15-mer peptides with 10 aa overlap. PreS1A and preS1B peptides refer to the consensus sequences (total 47 aa; region 2-48 aa), while OVA CTL and OVA Th at 10 µg/mL were used as negative controls. ConA was used as assay positive control at 0,5 µg/ml. Experimental vaccine cartoon was created with BioRender.com

Suppl. Figure 4. Prevention of HDV superinfection *in vivo*. Six human-liver chimeric mice transplanted with hepatocytes from donor L191501 were inoculated with patient-derived HBV and 8 weeks later, 4 out of 6 were passively immunized with 100 µl of mouse antiserum (end-point serum

titers: 10^5 on log scale) from D7/D8 mix protein prime-boost with QS21 adjuvant **(A)**. The remaining control mice received naïve mouse serum. One day later, all mice were HDV superinfected (patient-derived virus). The antiserum dosing schedule was repeated on day 1, 4 and 7 post-HDV superinfection with 50 µl (i.p.). **(B-C)** Show respectively HDV RNA and HBV DNA titers in mouse plasma. LOD (HDV) at 1:10 dilution is 187,5 IU/ml; LOD (HBV) at 1:10 dilution is 3.750 IU/ml. **(D-E)** Demonstrate respectively preS1A and preS1B antibody titers represented as end-point serum dilutions at which the OD value at 405 nm was at least twice the OD of the negative control (naïve mouse serum) at the same dilution. The cut-off was set at 60.
