# Supplementary figures and images for: Novel prime-boost immune-based therapy inhibiting both hepatitis B and D virus infections
Source: Gut. 2022 Aug 17;72(6):1186–95. doi: 10.1136/gutjnl-2022-327216 (PMC10176361; doi:10.1136/gutjnl-2022-327216)

Suppl. Figure 1. Characterization of protein constructs by SDS-PAGE and Western blot.

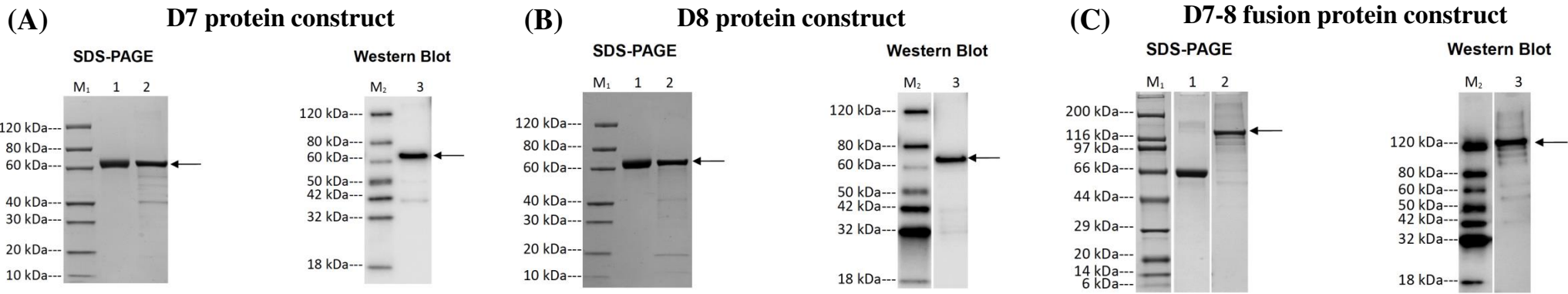

Supplement: Supplementary data [file gutjnl-2022-327216supp002.pdf]

Suppl. Figure 4. Prevention of HDV superinfection *in vivo*.

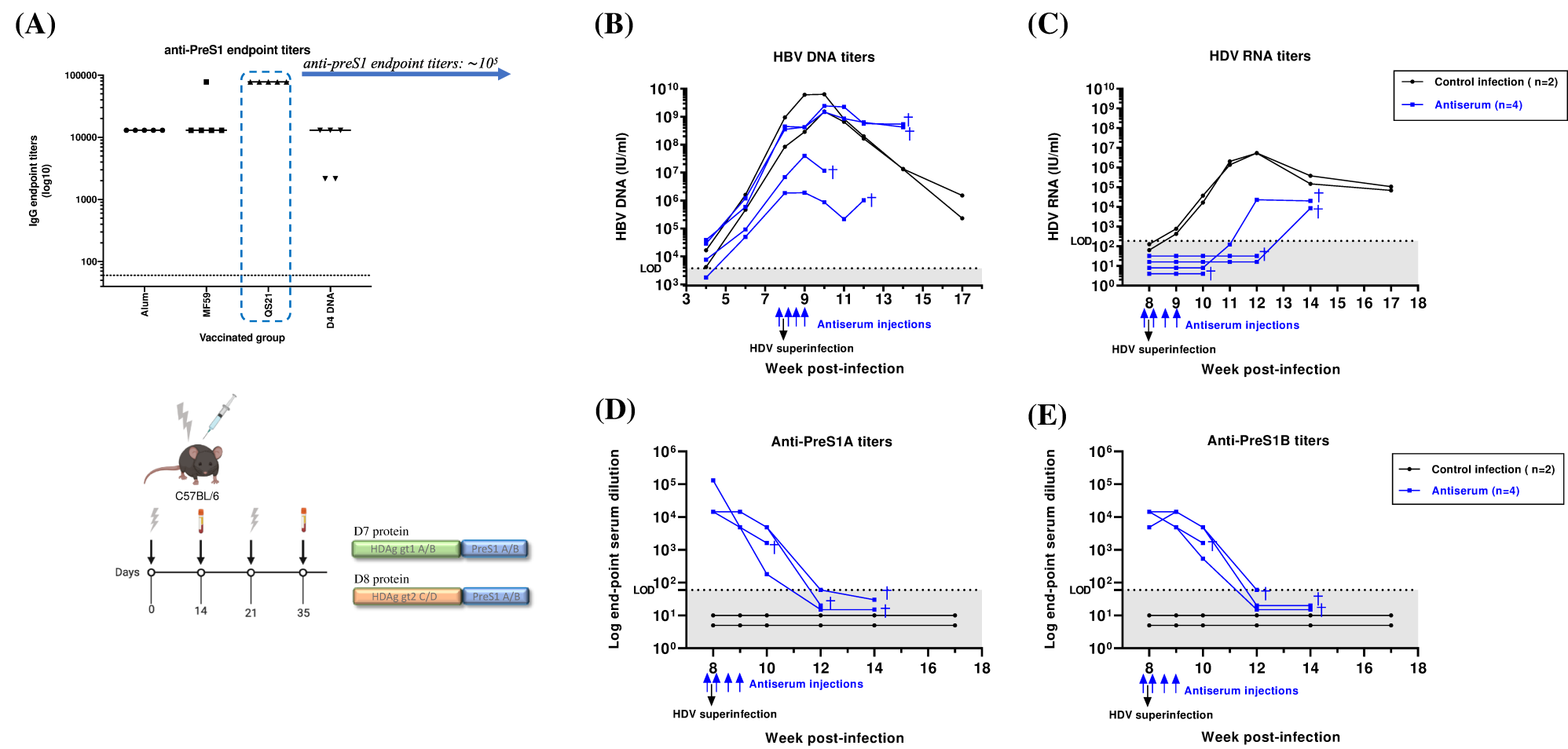

Supplement: Supplementary data [file gutjnl-2022-327216supp005.pdf]
