# Supplementary material for: Novel prime-boost immune-based therapy inhibiting both hepatitis B and D virus infections
Source: Gut. 2022 Aug 17;72(6):1186–95. doi: 10.1136/gutjnl-2022-327216 (PMC10176361; doi:10.1136/gutjnl-2022-327216)

Suppl. Figure 2. ELISA (A) and HBV *in vitro* neutralization (B) for all mice groups vaccinated with different combinations of DNA/protein preS1-HDAg or only HDAg constructs.

(A)

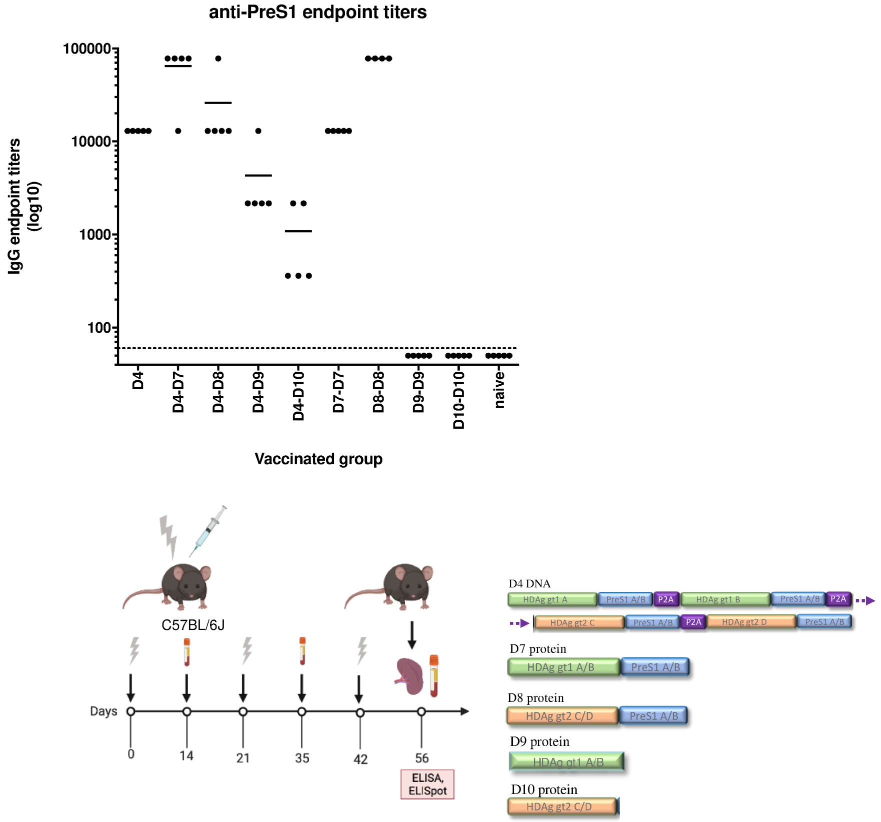

(B)

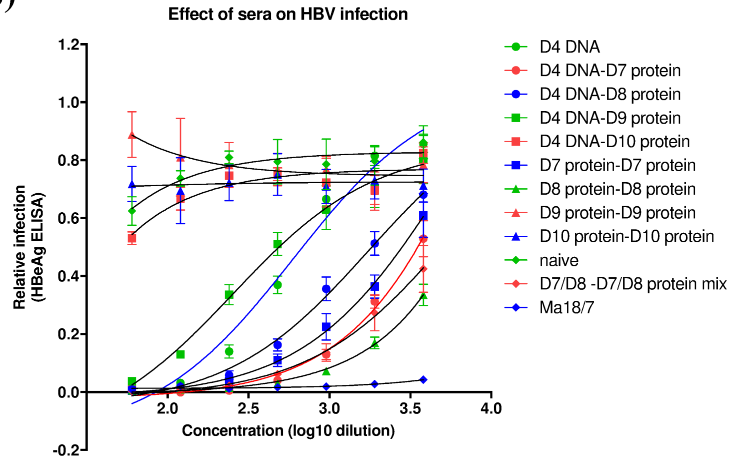

Supplement: Supplementary data [file gutjnl-2022-327216supp003.pdf]
