# Supplementary material for: Novel prime-boost immune-based therapy inhibiting both hepatitis B and D virus infections
Source: Gut. 2022 Aug 17;72(6):1186–95. doi: 10.1136/gutjnl-2022-327216 (PMC10176361; doi:10.1136/gutjnl-2022-327216)

# Suppl. Figure 3. Immunogenicity of heterologous prime-boost strategy based on D7-D8 fusion protein construct in C57BL/6 mice.

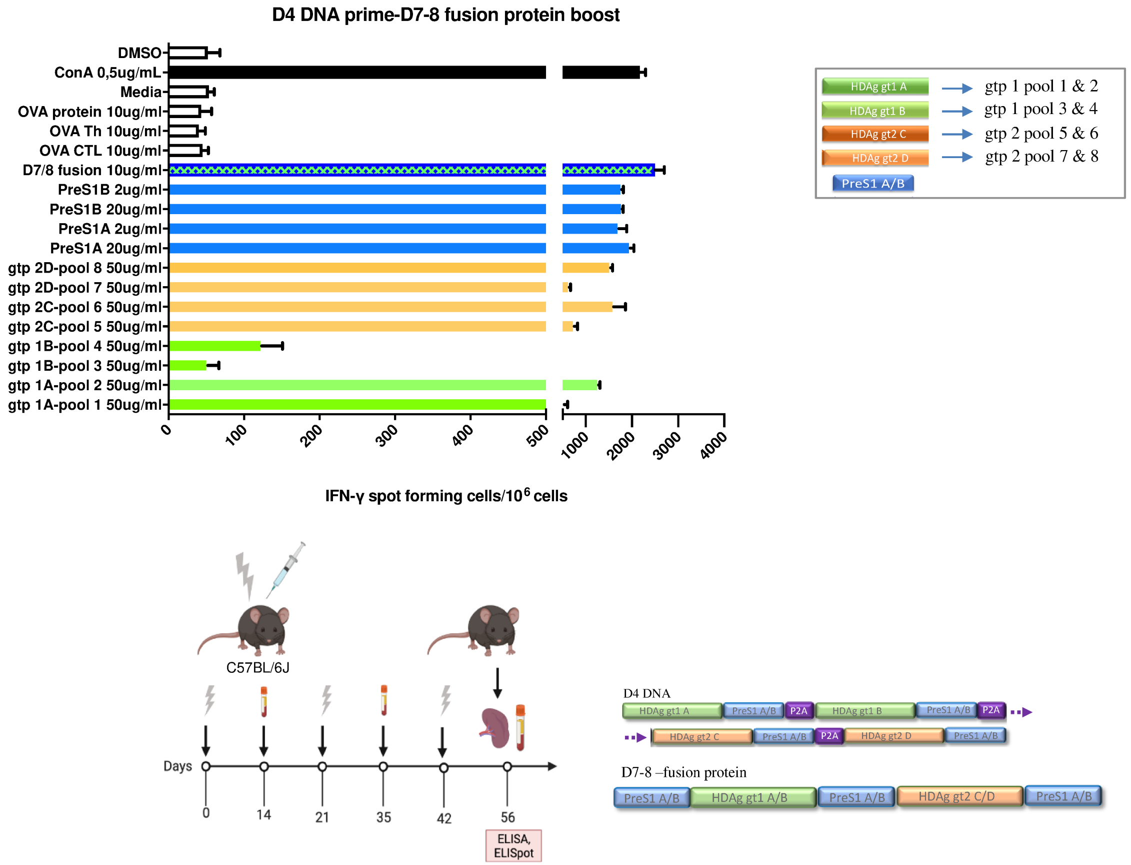

Supplement: Supplementary data [file gutjnl-2022-327216supp004.pdf]
